# Tolerance to intraoral biofilms and their effectiveness in improving mouth dryness and modifying oral microbiota in patients with primary Sjögren’s syndrome: “Predelfi study”

**DOI:** 10.3389/fmicb.2023.1071683

**Published:** 2023-02-08

**Authors:** Marie Orliaguet, Shao Bing Fong, Laëtitia Le Pottier, Vincent Meuric, Sylvie Boisramé, Martine Bonnaure-Mallet, Jacques-Olivier Pers

**Affiliations:** ^1^Univ Brest, CHU de Brest, Brest, France; ^2^University of Rennes 1, Rennes, France; ^3^LBAI, U1227, Univ Brest, Inserm, Brest, France

**Keywords:** Sjögren’s syndrome, mouth dryness, hyposialia, xerostomia, oral microbiota, prebiotic

## Abstract

**Introduction:**

Primary Sjögren’s syndrome (pSS) is a systemic autoimmune disease characterized by exocrine gland dysfunction. No therapeutic strategy is sufficient on its own for the management of dry mouth and therapeutic innovations are required.

**Methods:**

This Predelfi study was a single-center, prospective, comparative, randomized, double-blind, cross-over controlled study with the primary objective of assessing the tolerance to and effectiveness of two adhesive biofilms (containing prebiotics and, sodium alginate, respectively) in patients with pSS and hyposialia (#NCT04206826 in ClinicalTrials.gov). Secondary objectives were to obtain initial data regarding the clinical effectiveness of such biofilms in the improvement of signs and symptoms related to dry mouth and potential changes in the oral microbiota. Ten pSS patients with pSS were included (9 females and 1 male) with a mean age of 58.1 ± 14.0 years.

**Results and discussion:**

Tolerance to the prebiotic and sodium alginate biofilms was assessed by the patients (visual analog scale [VAS] score 66.7 and 87.6, respectively) and the practitioner (90 and 100, respectively). The absolute changes in the VAS scores at the start and end of each treatment period highlighted an improvement in mouth dryness for the sodium alginate versus the prebiotic biofilm. The VAS scores for other parameters (mouth burning sensation; taste alteration; chewing; swallowing and speech difficulties) remained globally comparable between the two groups. Unstimulated salivary flow showed no changes regardless of the biofilm used. Regarding the oral microbiota, the sodium alginate biofilm increased the abundance of the *Treponema* genus, whereas the use of the prebiotic biofilm as the first treatment increased the abundance of the genera *Veillonella* and *Prevotella*. Nevertheless, the prebiotic biofilm appeared to stimulate “milder” genera with regard to periodontal infections. Furthermore, pre-treatment with the prebiotic biofilm prevented the emergence of the *Treponema* genus induced by subsequent treatment with the sodium alginate biofilm, suggesting a potential protective effect.

## 1. Introduction

Xerostomia—the subjective feeling of dry mouth, and hyposialia—the objective and measurable decrease in salivary flow, affect at least a quarter of the population worldwide. This prevalence is higher in postmenopausal women and individuals over 65 years old (Edgar, 1990). Oral dryness may be physiological (related to age; Ship et al., 2002) or pathological (Thomson, 2005; López-Pintor et al., 2016) in nature. The most frequent pathological causes are the use of certain drugs, head and neck irradiation and pSS (Scully Cbe, 2003; Kielbassa et al., 2006; Thorne and Sutcliffe, 2017). Other factors such as depression, anxiety, stress, or malnutrition are also implicated in the etiology.

Primary Sjögren’s syndrome is a systemic autoimmune disease affecting 1—23 people per 10,000 inhabitants in European countries and occurs more frequently in women than in men, with a sex ratio of 9:1 (Cornec and Chiche, 2015; Brito-Zerón et al., 2016; Maciel et al., 2017). Dry mouth greatly handicaps patients in terms of their social life, as well as well-being by owing to its consequences in the oral cavity (Champey et al., 2006; López-Jornet and Camacho-Alonso, 2008; Milin et al., 2016). No therapeutic strategy is sufficient on its own for the management of dry mouth, and patients are awaiting therapeutic innovations in this area (Assery, 2019).

The oral microbiota includes all living microorganisms, that is, bacteria, viruses, archaea, and protozoa. Comprising over 700 different species of bacteria, the oral microbiota represents the second most diverse bacterial community in the human body (Aas et al., 2005; Dewhirst et al., 2010; Kilian et al., 2016). These different components, living in coexistence or in competition, form a complex microbial ecosystem, which is typically stable (Takahashi, 2005). However, an imbalance in this ecosystem due to various factors, including the oral environment and lack of immune response, leads to diseases such as dental caries, chronic periodontitis, and oral candidiasis (Marsh, 1994; Takahashi and Nyvad, 2011; Hebecker et al., 2014). One of the effects of hyposialia on the oral microbiota includes changes in the bacterial flora and salivary proteins. Hayashi et al. showed that hypo-salivation not only contributes to fluctuations in the number of certain microorganisms, but also influences the composition of the oral microbiota (microbial ecosystem; Hayashi et al., 2015). Rusthen et al. also reported dysbiosis in the salivary microbiota of patients with pSS and with dry mouth caused by other etiologies in comparison with healthy controls. Moreover, their findings suggest that the salivary microbiota in the pSS group and the non-pSS group differed significantly (Rusthen et al., 2019). Almståhl et al. reinforce this observation, as they highlight an increase in acidogenic and aciduric microorganisms in individuals with hyposalivation and changes in the oral microflora, which varied with the cause of hyposalivation (e.g., radiation-induced hyposialia, drug-induced hyposialia, and pSS; Almstahl and Wikstrom, 1999; Almståhl et al., 2001; AlmståhI et al., 2003; Almståhl and Wikström, 2005; Almstahl et al., 2008).

The main objective of this pilot study was to assess the tolerance and effectiveness of two adhesive biofilms (containing prebiotics and sodium alginate, respectively) in patients with pSS and hyposialia. Secondary objectives were to obtain initial efficacy data regarding the clinical effectiveness of such biofilms on the improvement of signs and symptoms related to dry mouth and potential modifications of oral microbiota.

## 2. Materials and methods

### 2.1. Study population

Ten patients with pSS were included. The Ethical Review Boards at Brest Hospital approved the study protocol (registration number NCT04206826, ClinicalTrials.gov). Diagnosis of pSS was made according to the ACR/EULAR classification criteria. Hyposialia was determined by measuring the stimulated salivary flow rate (SSF < 0.8 ml/min) and unstimulated salivary flow rate (USF < 0.3 ml/min).

### 2.2. Study design

**Figure 1:**
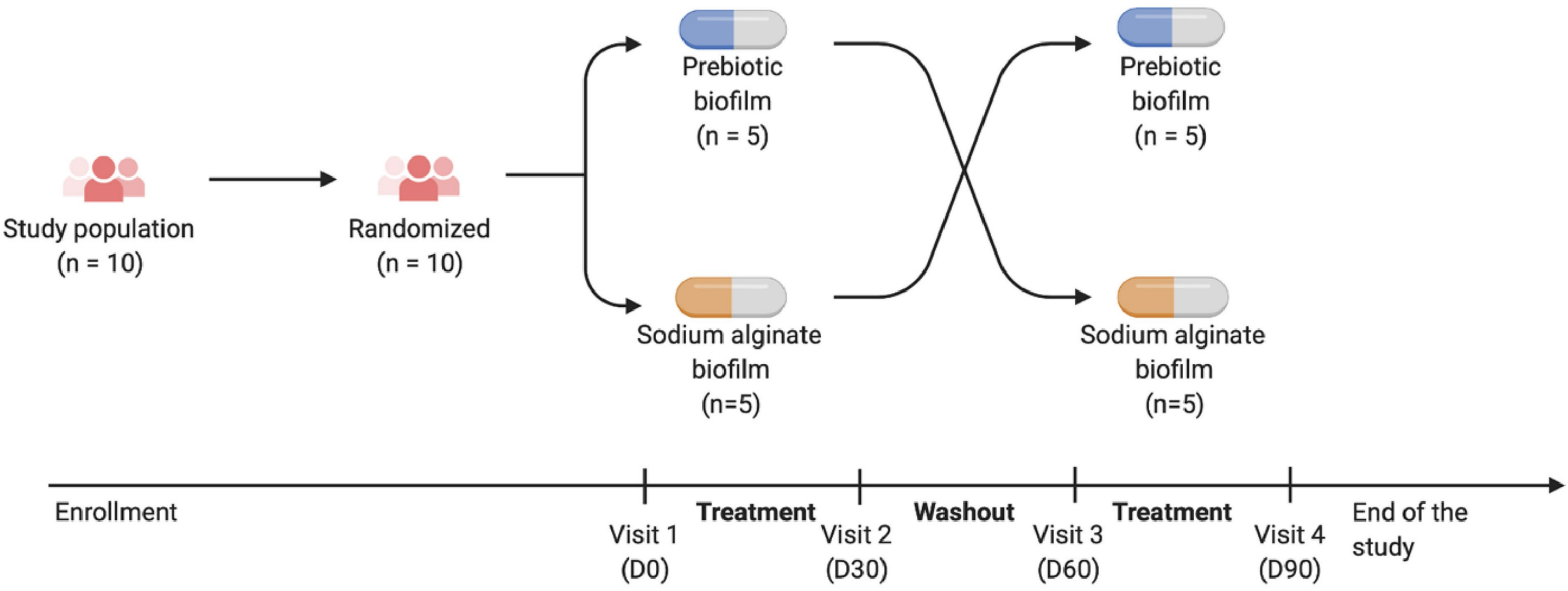
Study design.

The study was designed as a single-center, prospective, comparative, randomized, double-blind, cross-over controlled study. Each patient received a biofilm containing prebiotics (biofilm A) or sodium alginate (biofilm B) in a double-blind manner (operator and patient) for two treatment periods of 1 month interspersed with a 1-month washout period (Figure 1). The order of treatment (A then B or B then A) was assigned by randomization performed using the CSRandomization module of the Clinsight software. Half of the patients were randomly assigned to the group “A then B” and the other half to the group “B then A.”

### 2.3. Biofilms

Biofilm A comprised milk proteins, soy derivatives (alpha-oligosaccharide), vegetable glycerin, and water, whereas biofilm B included sodium alginate, water, glycerin, caramel, and *beta* carotene. Patients were required to apply the biofilm (one per day) upon waking in the morning after brushing teeth, on the gingival mucosa facing the maxillary vestibule. The patients were allowed to consume a drink until 5 min before the application but not for an hour after the application. The use of any type of mouthwash was to be avoided for the duration of the study.

The patients received a kit corresponding to each treatment period at the first visit (D0) and at the third visit (D60), including 30 biofilms (A or B) and a diary.

### 2.4. Clinical assessment

At each of the four visits (D0, D30, D60, and D90), data regarding the items listed in Table 1 were collected. Examination of the oral mucosa consisted of an objective clinical evaluation of the general state of the mucous membranes (e.g., redness, dryness, and degree of inflammation) with the establishment of a dry mouth clinical score (DMCS) based on the Challacombe scale (Osailan et al., 2011). In addition, at D30 and D90, the degree of redness and inflammation of the gingival mucosa near the site of application of the biofilm was assessed. The patients also completed a validated dry mouth self-report questionnaire comprising 18 items, and a self-report on specific signs and symptoms of dry mouth (such as slurred speech, chewing, swallowing, taste alterations, and burning sensations) using the VAS from 0 to 100 mm (Salom et al., 2015). As an example, 0 indicates no burning sensation and 100 indicates severe burning sensation.

**Table 1:** Elements collected during the different visits.

Actions	D-7 to D0	D30	Wash out	D60	D90
	(Inclusion visit and start of the first period)	(End of the first period)	(1 month)	(Start of the second period)	(End of the second period)study outing
Informed consent	X				
Validation of inclusion/exclusion criteria	X				
Medical history	X				
Concomitant treatments	X	X		X	X
Clinical examination of oral cavity and oral mucosa	X	X		X	X
Randomization	X				
Treatment dispensation (A or B)	X			X	
Dry mouth questionnaire (oral quality of life)	X	X		X	X
Tolerance assessment (VAS)		X			X
Saliva pH measurement	X	X		X	X
USF and SSF measurement	X	X		X	X
Storage of saliva (biocollection DC 2014–2,194)	X	X		X	X
Adverse events		X		X	X
Compliance monitoring		X			X

Stimulated and unstimulated salivary flow (SSF and USF).

Moreover, at each visit, SSF and USF rates were measured for a duration of 10 min without stimulation and 5 min with stimulation *via* paraffin chewing, respectively. The salivary pH was measured using a strip of pH paper.

The main evaluation criteria were tolerance to the biofilm at the end of the two treatment periods (D30 and D90), as assessed by the patients (VAS ranging from 0 to 100) and by the practitioner (degree of redness and inflammation of the gingival mucosa near the site of application of the biofilm) and self-reported adverse events related to the biofilm use.

The secondary evaluation criteria were self-assessment of oral comfort on a VAS of 0–100 mm (measurement of absolute variation at the start and end of each treatment period; absolute changes in specific signs and symptoms of dry mouth by subjective evaluation of mouth dryness; mouth burning sensation; taste alteration; chewing; swallowing, and speech difficulties, on a VAS of 0–100 mm), absolute changes in SSF and USF rates, changes in oral pH using pH paper, and alterations in the salivary microbiota assessed by molecular sequencing.

### 2.5. Sample DNA extraction and amplification of bacterial DNA

Saliva samples were collected in tubes, and total DNA was extracted using the DNeasy® Blood and Tissue Kit (Qiagen) according to the manufacturer’s instructions. The DNA was stored at −80°C until further use. PCR amplification of DNA was performed using PuReTaq™ Ready-To-Go™ PCR beads (Cytiva) according to the manufacturer’s instructions. The V1–V3 regions of the 16S rRNA gene were amplified using the primers 8F (5′-AGA-GTT-TGA-TCC-TGG-CTC-AG-3′) and 534R (5′-ATT-ACC-GCG-GCT-GCT-GG-3′) with 25 cycles of PCR at an annealing temperature of 60°C. The ZymoBIOMICS Microbial Community DNA Standard (Zymo Research) was included in the amplification repertoire as a positive control. All PCR products were resolved by electrophoresis on a 2% agarose gel in tris-acetate-EDTA buffer to confirm amplification, and subsequently sequenced with the Illumina MiSeq at the EcogenO facility (Rennes University, France).

### 2.6. Oral microbiota analysis

The FASTQ files were processed with the QIIME2 software (v. 2021–8, https://qiime2.org/; Bolyen et al., 2019) and were imported in the “PairedEndFastqManifestPhred33”-format. The pipeline DADA2 was used to control the sequence quality and construct the feature table (Callahan et al., 2016).The forward and reverse sequences were truncated at 300 and 280 bases, respectively, with all other parameters set to default. Sequence count per sample ranged between 2,298 and 15,225. Prior to taxonomic assignment, reference reads were extracted using the 16S rRNA reference sequences (HOMD_16S_rRNA_RefSeq_V15.22. p9.fasta) obtained from the expanded Human Oral Microbiome Database V3 website (https://www.homd.org; eHOMD), on the basis of matches to the primer pair (8F/534R). The resulting reference reads were then trained as a Naïve Bayes classifier with the corresponding eHOMD 16S rRNA reference sequence taxonomy file for QIIME (HOMD_16S_rRNA_RefSeq_V15.22. qiime.taxonomy) that was also obtained from the eHOMD website. Core diversity analyses included *alpha* (Faith’s Phylogenetic Diversity; Faith, 1992) and *beta* diversity metrics (weighted UniFrac, and PCoA Bray–Curtis; Bray and Curtis, 1957; Lozupone et al., 2007). Each of the feature tables with taxonomic assignment at the species level was exported for further analyses. Sequence data will be made available upon request.

### 2.7. Statistical analysis

Quantitative variables were expressed as mean and SD, and qualitative variables were expressed as absolute value and percentage.

Although this is a primarily descriptive and non-confirmatory pilot study, the cross-over design has been classically analyzed using a mixed model including the factors treatment (prebiotic or sodium alginate biofilm, fixed effect), period (first or second, fixed effect), and order (prebiotic or sodium alginate then sodium alginate or prebiotic biofilms, fixed effect) as well as the subject factor (random effect) nested in the order factor. For exploratory purposes, the degree of significance was set at 5%.

Microbiome bioinformatics data were processed using the QIIME 2 pipeline (Bolyen et al., 2019). Non-parametric tests were used and considered significant at *p* < 0.05. The results were expressed as mean ± standard error of the mean. The Kruskal–Wallis pairwise test with Benjamini–Hochberg False Discovery Rate (FDR) correction was used to compare means for qualitative data related to *alpha* diversity indices (Benjamini and Hochberg, 1995). The PERMANOVA test was performed on *beta* diversity metric using the QIIME2 diversity plugin. A linear discriminant analysis of the relative abundance of taxa was performed with the linear discriminant analysis effect size (LEfSe) algorithm, where all parameters were set to default in the Galaxy web application.^1^ All plots were generated through QIIME2 and Galaxy (Segata et al., 2011; Vázquez-Baeza et al., 2013).

## 3. Results

### 3.1. Patient characteristics at baseline

Ten patients with pSS (one male and nine females) were included in the study, with a mean age of 58.10 ± 14.04 years. One patient reported depressive symptoms and four reported certain thyroid problems. No patient received treatment with drugs known to induce xerostomia. Two patients were ex-smokers and eight had never smoked. The mean number of alcoholic drinks consumed per day was 0.20 ± 0.42.

They were randomized into two arms of equal size: prebiotic biofilm (A) then Sodium alginate biofilm (B) group (*n* = 5) and sodium alginate biofilm (B) then Prebiotic biofilm (A) group (*n* = 5). All patients completed the study in their initial randomization group, with no missing data (Supplementary Figure 1).

Assessment of the clinical parameters at inclusion are shown in Table 2. The average USF and SSF rates were 0.3 ± 0.25 and 0.6 ± 0.39 ml/min, respectively, and the average pH was 7.3 ± 0.95. The average dry mouth clinical score, based on the Challacombe scale, was 4.7 ± 1.42, which corresponded to a moderately dry mouth. The initial clinical examination performed by the practitioner did not indicate any inflammation with normal oral mucosa in 90% of cases (*n* = 9) and mild inflammation in 10% of cases (*n* = 1). Furthermore, no erythema of the oral mucosa was observed in 80% of cases (*n* = 8), and mild erythema was observed in 20% of cases (*n* = 2). No patient presented oral candidiasis, and only one patient exhibited trauma of the oral mucosa. Regarding the self-assessment, the average VAS score for dry mouth was 58.4 ± 17.9 mm (range = 35–90 mm).

**Table 2:** Clinical parameters at inclusion (D0).

Variable	Global population (*n* = 10)	Arm 1: Prebiotic biofilm A (*n* = 5)	Arm 1: Sodium alginate biofilm B (*n* = 5)
USF (ml/min), mean +/− SD	0.3 ± 0.25	0.2 ± 0.19	0.4 ± 0.29
SSF (ml/min), mean +/− SD	0.6 ± 0.39	0.7 ± 0.44	0.5 ± 0.36
Salivary pH, mean +/− SD	7.3 ± 0.95	7.8 ± 0.45	6.8 ± 1.09
DMCS (Challacombe scale), mean +/− SD	4.7 ± 1.42	5.2 ± 1.48	4.2 ± 1.30
Inflammation of the oral mucosa, *n* (%)			
Normal	9 (90%)	4 (80%)	5 (100%)
Mild	1 (10%)	1 (20%)	0
Moderate	0	0	0
Severe	0	0	0
Erythema of the oral mucosa, *n* (%)			
Normal	8 (80%)	4 (80%)	4 (80%)
Mild	2 (20%)	1 (20%)	1 (20%)
Moderate	0	0	0
Severe	0	0	0
Halitosis/fetid breath, *n* (%)			
Normal	8 (80%)	4 (80%)	4 (80%)
Mild	0	0	0
Moderate	2 (20%)	1 (20%)	1 (20%)
Severe	0	0	
Speech difficulties, *n* (%)			
Normal	5 (50%)	2 (40%)	3 (60%)
Mild	4 (40%)	2 (40%)	2 (40%)
Moderate	1 (10%)	1 (20%)	0
Severe	0	0	0
Oral mucosal adhesion, *n* (%)			
Normal	1 (10%)	0	1 (20%)
Mild	5 (50%)	2 (40%)	3 (60%)
Moderate	4 (40%)	3 (60%)	1 (20%)
Severe	0	0	0
Foamy state of saliva, *n* (%)			
Normal	6 (60%)	2 (40%)	4 (80%)
Mild	4 (40%)	3 (60%)	1 (20%)
Moderate	0	0	0
Severe	0	0	0
VAS (0–100 mm), mean +/− SD and (min–max)			
VAS mouth dryness (mm)	58.4 ± 17.87 (35.0–90.0)	61.8 ± 14.65 (45.0–80.0)	55 ± 21.79 (35.0–90.0)
VAS mouth burning sensation (mm)	19.8 ± 30.29 (0–87.0)	6.2 ± 13.86 (0–31.0)	33.4 ± 37.55 (0–87.0)
VAS taste alteration (mm)	16.5 ± 24.47 (0–70,0)	16.6 ± 30.13 (0–70.0)	16.4 ± 20.96 (0–46.0)
VAS chewing difficulties (mm)	16.1 ± 27.70 (0–90.0)	4.8 ± 8.67 (0–20.0)	27.4 ± 36.49 (0–90.0)
VAS swallowing difficulties (mm)	23.8 ± 28.61 (0–71.0)	40.8 ± 31.95 (0–71.0)	6.8 ± 9.93 (0–24.0)
VAS speech difficulties (mm)	13.5 ± 19.80 (0–61.0)	17.8 ± 25.94 (0–61.0)	9.2 ± 12.77 (0–26.0)

SSF, stimulated salivary flow; USF, unstimulated salivary flow; DMCS, dry mouth clinical score; VAS, visual analog scale; Min, minimum; and Max, maximum.

Supplementary Table 1 summarizes the responses to the self-questionnaire filled at the inclusion visit (D0). In total, 60% of the patients reported frequent dry mouth episodes, 40% reported constant nocturnal awakenings with the need to drink to reduce xerostomia, 60% complained of sometimes having thick saliva, and 80% never or sometimes experienced pain in the mouth. Regarding the chewing and swallowing aspect, 50% of the patients never had a sore throat and 50% sometimes had a sore throat when swallowing. Moreover, 50% of the patients never had difficulties in chewing and/or swallowing solid food, whereas 40% frequently or always experienced those problems. Furthermore, 70% of the patients sometimes or often needed to take sips of a liquid to swallow food, and 60% of the patients had no difficulty enjoying meals, whereas 60% had difficulties carrying on a conversation without stopping to drink.

### 3.2. Changes in subjective and objective parameters vary between D0 (inclusion) and D60 (wash-out)

**Figure 2:**
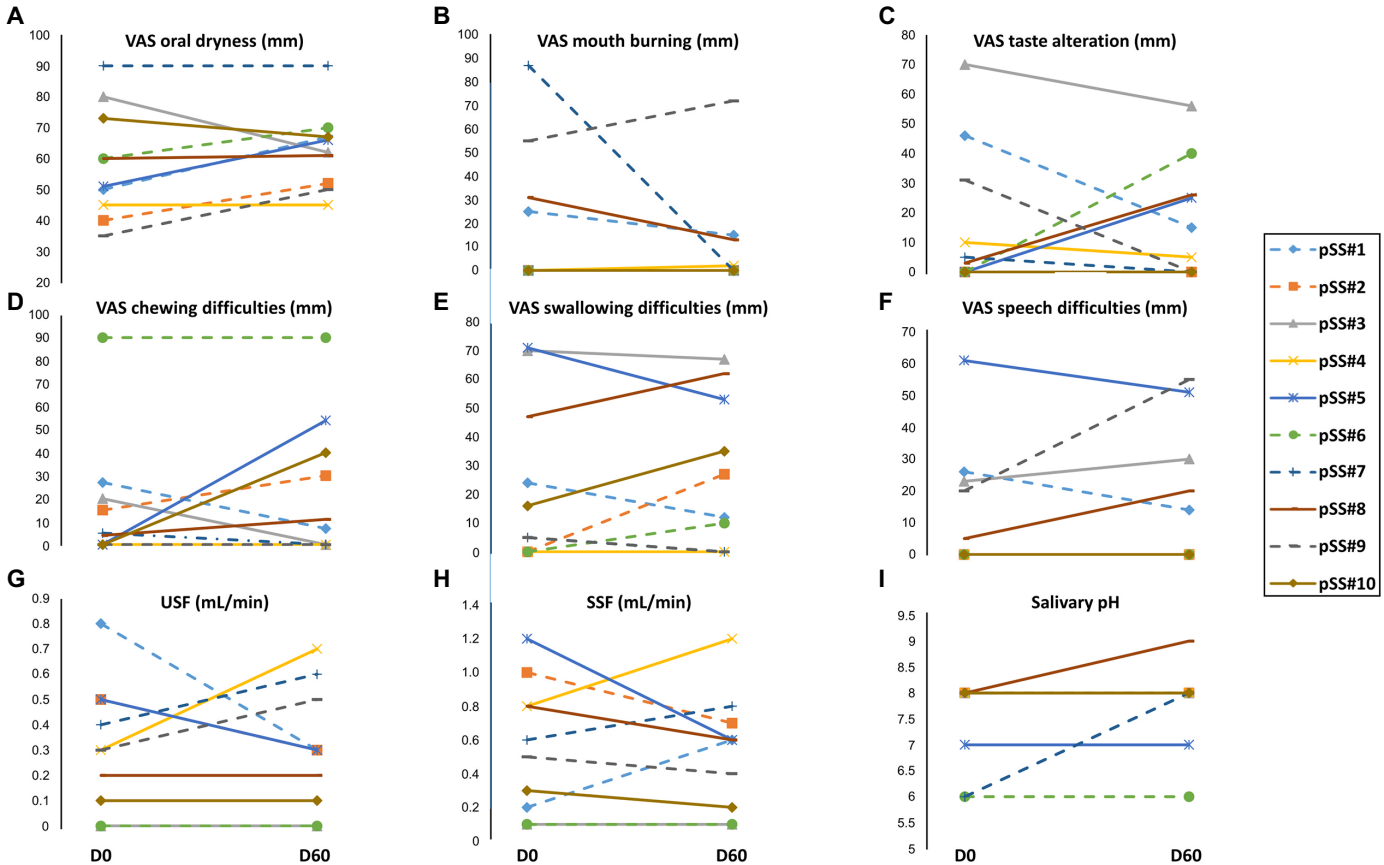
Subjective [visual analog scale (VAS) scores] and objective evaluation of oral dryness between baseline D0 and D60 (1 month after wash-out). **(A)** VAS scores for dry mouth, **(B)** VAS scores for mouth burning, **(C)** VAS scores for taste alteration, **(D)** VAS scores for chewing difficulties, **(E)** VAS scores for swallowing difficulties, **(F)** VAS scores for speech difficulties, **(G)** Unstimulated salivary flow (USF) rate, **(H)** Stimulated salivary flow (SSF) rate, and **(I)** Salivary pH.

Analysis of subjective criteria assessed on a VAS (oral dryness, taste alteration, chewing, and swallowing and speech difficulties) at inclusion (D0) and at the end of the wash-out period (D60) showed changes (Figure 2). Notably, the oral dryness VAS score of patients who received alginate sodium biofilm B in the first period (patients 1, 2, 6, 7, and 9, -dotted curves-in Figure 2A) tended to increase from D0 to D60 (Figures 2B–F). This observation demonstrates that VAS score in patients with pSS fluctuated with time.

The USF rate (Figure 2G) was not significantly different between D0 and D60 in four patients (#3, #6, #8, and #10), showed an increase in three patients (#4, #7, and #9), and showed a decrease in the three remaining patients (#1, #2, and #5). The SSF rate (Figure 2H) was not significantly different between D0 and D60 in only two patients (#3 and #6). For the other patients, upward or downward fluctuations in the SSF rate unrelated to the treatment received in the first period were noted. An increase in the salivary pH from D0 to D60 (Figure 2I) was observed for two patients (#7 and #9).

### 3.3. Assessment of tolerance to the two biofilms

The tolerance of patients to the two biofilms during the different treatment periods is shown in Table 3. During the first treatment period, the sodium alginate (B)-prebiotic (A) arm estimated the tolerance to sodium alginate biofilm B at 80.6 ± 20.9, whereas the prebiotic (A)-sodium alginate (B) arm estimated the tolerance to prebiotic biofilm at 87.6 ± 22.6. However, during the second treatment period, the sodium alginate (B)-prebiotic (A) arm estimated the tolerance to prebiotic biofilm A at 45.8 ± 33.1, whereas the prebiotic (A)-sodium alginate (B) arm estimated the tolerance to sodium alginate biofilm B at 94.6 ± 7.4. Although the difference between the two means was not statistically significant, it appeared that the order in which treatments were received was a determinant and suggested a higher acceptability of sodium alginate biofilm (B).

**Table 3:** Analysis of tolerance over the two periods.

Variable		Sodium alginate biofilm	Prebiotic biofilm	p^*^
Patient tolerance VASPeriod 1	*n*	5	5	
	Mean +/− SD	80.6 ± 20.9	87.6 ± 22.6	0.656^*^
	Min–Max	50;100	48;100	
Patient tolerance VASPeriod 2	*n*	5	5	
	Mean +/− SD	94.6 ± 7.4	45.8 ± 33.1	0.067^*^
Min–Max		86;100	10;100	

VAS, visual analog scale; Min, minimum; and Max, maximum.

* Test de Mann–Whitney (Wilcoxon).

Regarding the assessment of the biofilm placement site by the practitioner, only one patient showed inflammation with the prebiotic biofilm A in the first period, and it was due to a temporary trauma of the mucosa because of the rigidity of the biofilm. The tolerance to the prebiotic biofilm assessed by the practitioner was therefore 90 and 100% for prebiotic biofilm (A) and the sodium alginate biofilm (B), respectively.

### 3.4. Assessment of subjective and objective parameters according to treatment

Analysis of the absolute changes in the VAS scores at the beginning and end of each treatment period is shown in Table 4. The most significant result was an increase in the VAS score for mouth dryness of 10.8 ± 12.8 mm for sodium alginate biofilm B versus 0.9 ± 17.8 mm for prebiotic biofilm A. The VAS score for mouth burning sensation increased by 6.3 ± 29.2 mm with sodium alginate biofilm B and by 9.2 ± 22.2 mm for prebiotic biofilm A. The VAS scores for taste alteration and chewing difficulties remained globally comparable within the two groups. The VAS scores for swallowing and speech difficulties were improved with prebiotic biofilm A (7.9 ± 18.2 and 6.2 ± 10.6 mm, respectively) whereas no change was observed with sodium alginate biofilm B. Individual data according to the randomization group highlight the changes more precisely (Supplementary Figure 2). No change was observed in USF, and SSF rates and salivary pH regardless of the biofilm used (data not shown).

**Table 4:** Absolute change of start-end VAS of each treatment period.

Variable		Sodium alginate biofilm (*n* = 10)	Prebiotic biofilm (*n* = 10)
VAS mouth dryness: absolute change beginning-end of each treatment period	Mean +/− SD	−10.8 ± 12.78	−0.9 ± 17.80
	Min–Max	−30.0;15.0	−26.0;39.0
VAS mouth burning sensation: absolute change beginning-end of each treatment period	Mean +/− SD	6.3 ± 29.21	9.2 ± 22.07
	Min–Max	−27.0;60.0	−5.0;70.0
VAS taste alteration: absolute change beginning-end of each treatment period	Mean +/− SD	0.9 ± 11.79	1.2 ± 5.12
	Min–Max	−26.0;18.0	−5.0;14.0
VAS chewing difficulties: absolute change beginning-end of each treatment period	Mean +/− SD	3.9 ± 29.48	1.9 ± 17.91
	Min–Max	−23.0;80.0	−30.0;42.0
VAS swallowing difficulties: absolute change beginning-end of each treatment period	Mean +/− SD	−0.5 ± 12.89	−7.9 ± 18.25
	Min–Max	−20.0;24.0	−54.0;7.0
VAS speech difficulties: absolute change beginning-end of each treatment period	Mean +/− SD	−2.1 ± 8.10	−6.2 ± 10.58
	Min–Max	−21.0;10.0	−33.0;1.0

VAS, visual analog scale; Min, minimum; and Max, maximum.

### 3.5. *Alpha* and *beta* community diversity

**Figure 3:**
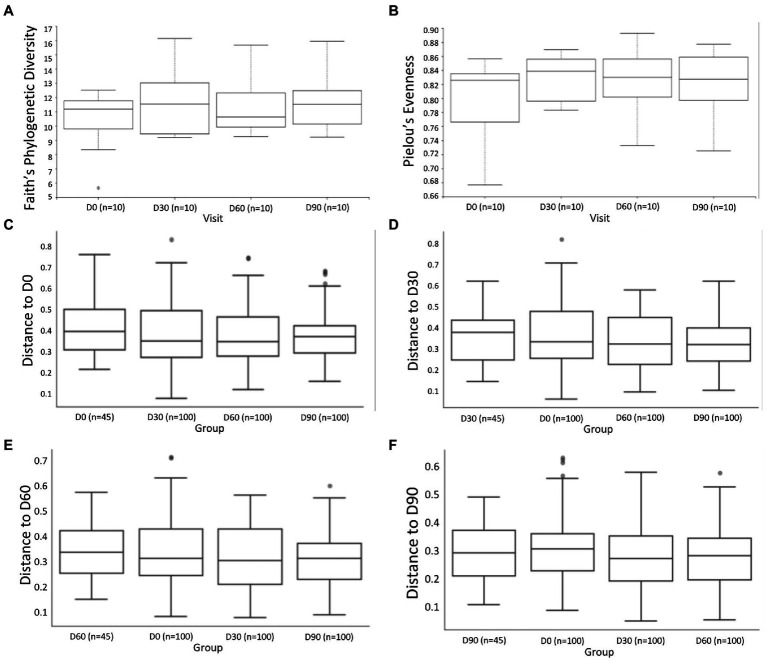
*Alpha* diversity metrics comparing community **(A)** richness and **(B)** evenness between study visits, respectively. Kruskal–Wallis pairwise comparisons with Benjamini–Hochberg False Discovery Rate (FDR) correction were performed. *Beta* diversity metrics included Weighted UniFrac **(C–F)** with PERMANOVA test (999 permutations) comparing community dissimilarity across study visits.

To assess the community diversity between the treatment time-points and among participants, *alpha* and *beta* diversity metrics were estimated in the QIIME2 pipeline. The *alpha* diversity metrics Faith’s phylogenetic diversity and Pielou’s evenness showed that there was no significant difference in richness and evenness between study visits (Figures 3A,B). The *beta* diversity metric weighted UniFrac indicated no significant difference in community dissimilarity between study visits were found (Figures 3C–F).

**Figure 4:**
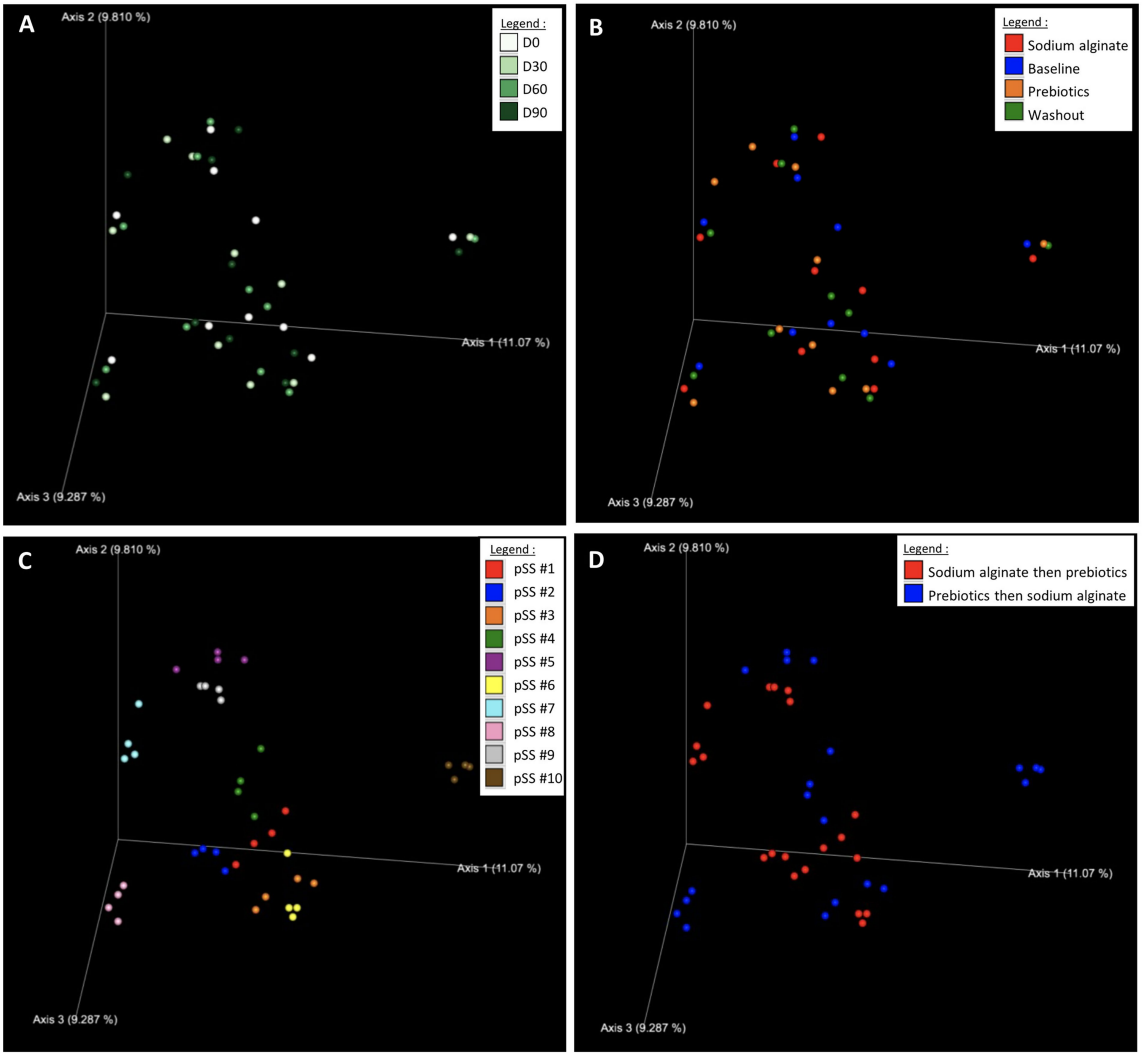
*Beta* diversity metrics Bray–Curtis Emperor plots for **(A)** study visits, **(B)** study phases, **(C)** participants, and **(D)** treatment sequence. The legend colors indicate the respective sample identities.

The Bray–Curtis dissimilarity emperor plots showed that study visits (Figure 4A), and study phases (Figure 4B) were clustered by participants (Figure 4C). The participants were also differentiated according to their respective treatment group (Figure 4D), and were well dispersed over the three axes displayed. Although a distinct pattern or clustering was absent (apart from clustering by participants), a slight shift from the reported baseline was observed for certain individuals (Figures 4B,C). This observation is not specific for either treatment sequence group.

### 3.6. Microbial taxonomy

Taxonomic classification was performed and presented as relative frequencies in a bar chart (Supplementary Figure 3). The 20 most abundant bacterial taxa in all samples are listed and color-coded in the figure, including the genera *Streptococcus*, *Neisseria,* and *Veillonella*, and species *Haemophilus parainfluenzae*, *Veillonella atypica, Prevotella melaninogenica*, and *Porphyromonas pasteri*. As the bar chart denotes, each patient presented a different microbial composition and slight alterations in relative frequencies were observed through the study phases. However, no significant changes were observed after each treatment, which mirrors the earlier Bray–Curtis results. Supplementary Table 2 presents a list of bacterial species and their relative frequencies across the samples.

### 3.7. Differential abundance analysis by linear discriminant analysis effect size

**Figure 5:**
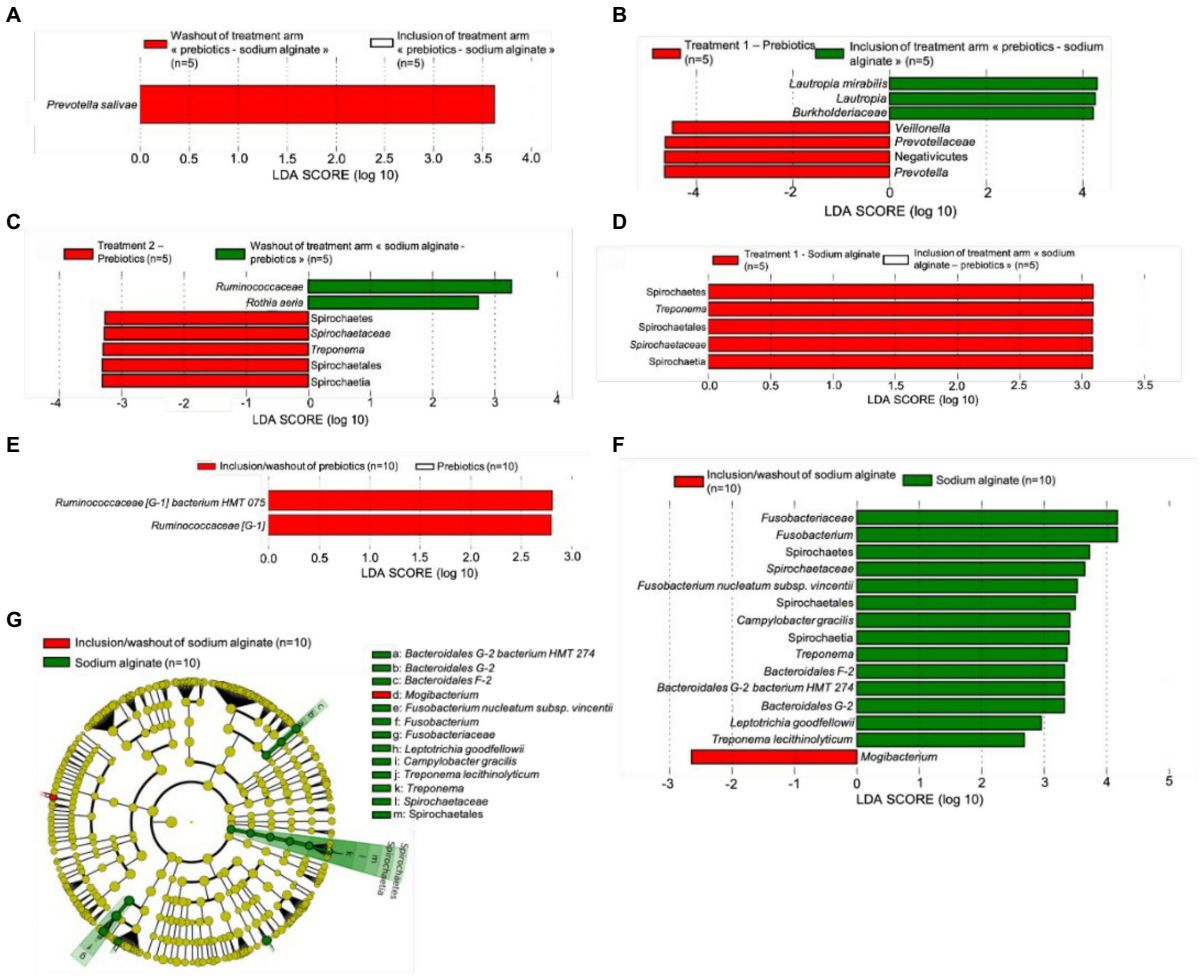
Linear discriminant analysis effect size (LEfSe) of significant taxa **(A)** with a different relative abundance between inclusion and washout time points of the treatment arm “prebiotic biofilms-sodium alginate biofilm,” **(B)** indicating effect of the prebiotic biofilm in the first phase (D0 versus D30), **(C)** indicating effect of the prebiotic biofilm in the second phase (D60 versus D90), **(D)** indicating effect of the sodium alginate biofilm in the first phase (D0 and D30), **(E)** indicating overall effect of the prebiotic biofilm from the start to the end of study, **(F)** indicating overall effect of the sodium alginate biofilm from the start to the end of study, and **(G)** cladogram representing the overall effect of the sodium alginate biofilm from the start to the end of study. The length of the bar represents a log10 transformed LDA score, which was computed by the algorithm by the Galaxy web application.

The analyses described above suggested that the microbial community of each participant responds to the treatment phases differently, and thus, it would be difficult to discern whether there is a real effect. Despite the lack of significant differences observed in the diversity analyses, the taxonomic data at the species level was further analyzed using the LEfSe algorithm. The effect size (linear discriminant analysis; LDA) of the taxa with significantly different abundance between the groups are presented in Figure 5.

To analyze the microbiome of the study population (*n* = 10), the data were divided into their respective groups: the prebiotic biofilm (A) then sodium alginate biofilm (B) group (*n* = 5) and the sodium alginate biofilm (B) then prebiotic biofilm (A) group (*n* = 5). The microbiome at baseline was first compared between the two groups and no differentially abundant features was observed (data not shown). The microbial population of each treatment arm at D60 (after the washout) was then assessed to examine whether it differed compared with the microbial population at D0. No differentially abundant features were reported in sodium alginate biofilm (B) then prebiotic biofilm (A) treatment group, whereas *Prevotella salivae* was found to have higher abundance at D60 than D0 in prebiotic biofilm (A) then sodium alginate biofilm (B) treatment group (Figure 5A).

Moreover, the effect of the prebiotic biofilm (A) and sodium alginate biofilm (B) in the first treatment period (D0 versus D30) and second treatment period (D60 versus D90) was assessed. When prebiotic biofilm (A) was administered in the first treatment period, an increase in relative abundance was observed for the genera *Prevotella* (family *Prevotellaceae* and order Bacteroidales) and *Veillonella* (class Negativicutes; Figure 5B). When prebiotic biofilm (A) was administered in the second treatment period, a significant increase in relative abundance was observed for the genus *Treponema* (family *Spirochaetaceae*, order Spirochaetales and class Spirochaetia; Figure 5C). When sodium alginate biofilm (B) was administered in the first treatment period, an increase in relative abundance was observed for the genus *Treponema* (Figure 5D), similar to the trend observed when prebiotic biofilm (A) was used as the second treatment. However, no differentially abundant features were found when sodium alginate biofilm (B) was administered in the second treatment period. These findings suggest that the order of the treatment influenced the final outcome of the study. For example, using sodium alginate (B) as the first treatment increased the abundance of the *Treponema* genus. This effect persisted in the second treatment period during which prebiotic biofilm (A) was administered. Notably, *Lautropia mirabilis* (genus *Lautropia*, family *Burkholderiaceae*) showed a higher relative abundance at inclusion, and, *Rothia aeria* and family *Ruminococcaceae* showed a higher relative abundance at the washout (D90), prior to treatment with prebiotic biofilm (A).

When the absolute effect of each biofilm treatment was assessed, *Ruminococcaceae [G-1] bacterium HMT 075* showed a higher relative abundance during non-treatment phases (D0 and D90) than during treatment with prebiotic biofilm (A; Figure 5E). However, when the absolute effect of sodium alginate biofilm (B) was assessed (Figure 5F), *Fusobacterium nucleatum subsp. vincentii* (family *Fusobacteriaceae*), *Treponema lecithinolyticum* (family *Spirochaetaceae*, order Spirochaetales, class Spirochaetia and phylum Spirochaetes), *Bacteroidales [G-2] bacterium HMT 274* (from the genus *Bacteroidales [G-2]*), *Bacteroidales [F-2]* genus, *Campylobacter gracilis* and *Leptotrichia goodfellowii* showed a higher relative abundance in the treatment phases than in the non-treatment phases. Only the *Mogibacterim* genus showed a higher relative abundance in the non-treatment phases than during treatment with sodium alginate biofilm (B; Figure 5F). Figure 5G shows a cladogram demonstrating the effects of sodium alginate biofilm (B).

**Figure 6:**
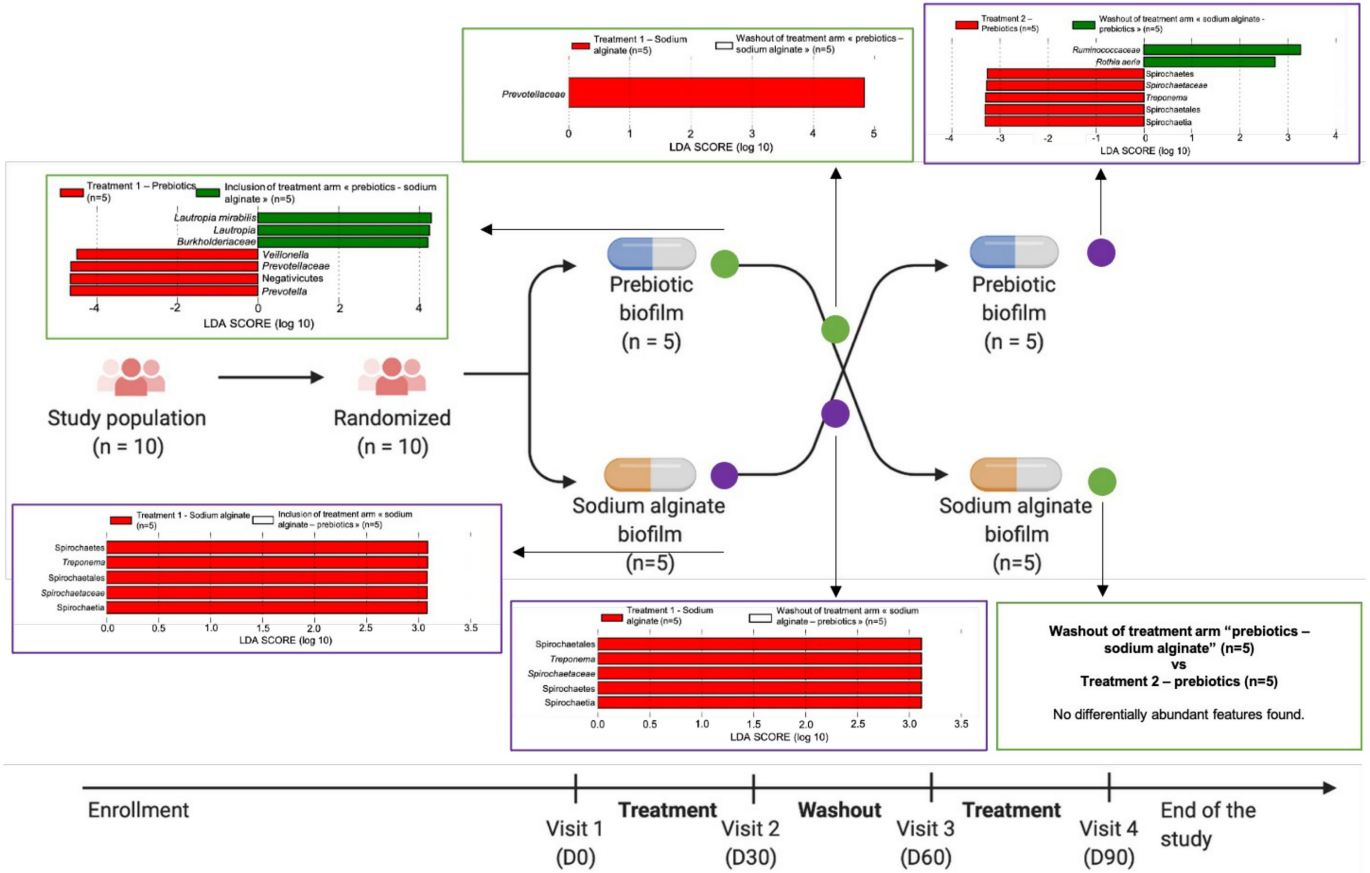
Linear discriminant analysis effect size (LEfSe) charts showing the evolution of the oral microbiota in each phase over the study period. Green circles represent the treatment arm “prebiotic biofilm—sodium alginate biofilm” and purple circles represent the treatment arm “sodium alginate biofilm—prebiotic biofilm.”

The findings showed that each biofilm had a distinct effect on the evolution of the oral microbiota in each treatment arm, in each treatment phase over the study period (Figure 6). When prebiotic biofilm (A) was administered as the first treatment, a higher relative abundance of *Prevotella* and *Veillonella* was observed. *Prevotellaceae* showed a lower relative abundance in the washout phase than in the first treatment period with prebiotic biofilm (A), and no difference was observed after the second treatment with sodium alginate biofilm (B). In contrast, when administered as the first treatment, sodium alginate biofilm (B) promoted an increase in the relative abundance of the *Treponema* genus. The increase persisted even after prebiotic biofilm (A) administration as the second treatment. Taken together, these results suggest that prebiotic biofilm (A) protected against the effects of sodium alginate biofilm (B), which appeared to promote the growth of the pathogenic genus *Treponema*. However, administrating prebiotic biofilm (A) as the second treatment was not sufficient to counter the effect of sodium alginate biofilm (B) as the first treatment.

## 4. Discussion

The main objective of this study was to evaluate the tolerance to adhesive biofilms containing prebiotics and sodium alginate in patients with pSS and hyposialia. The secondary objectives were to obtain initial efficacy data regarding the clinical effectiveness of such biofilms in improvement of signs and symptoms related to dry mouth and to assess potential changes in the oral microbiota.

Tolerance to prebiotic and sodium alginate biofilms was assessed by the patients (VAS 66.7 and 87.6, respectively) and by the practitioner (90 and 100%, respectively). Estimation of the absolute changes in the VAS scores at the beginning and end of each treatment period highlighted an improvement in the VAS for mouth dryness for the sodium alginate biofilm versus prebiotic biofilm, while no difference in the VAS scores was observed for other parameters.

Several limitations to the study may be highlighted. First, the potential bias due to the difference in characteristics between the two groups is removed by the study design. Indeed, the cross-over design makes it possible to consider each patient as their own control and thus ensures the comparability of the two groups despite the small number and the potential differences. Second, during the statistical analysis of tolerance, a significant follow-up effect at 10% was found. The statistical model therefore does not allow us to conclude that the treatment explains the difference in tolerance between the two groups; the order in which the treatments were received would explain part of the difference. The results of the second period can only be used to understand the data and formulate hypotheses and not to draw conclusions from the study. Third, a difference in the characteristics of patients between inclusion (D0) and the end of the wash-out period (D60) can be observed. The effects of the biofilm during the second treatment period cannot therefore be compared with those observed during the first period. The washout period may be extended, but as shown in Supplementary Figure 2, the fluctuations in the values of parameters between D30 and D60 are not necessarily consistent with the changes observed in the first period (D0–D30). This difference in characteristics between inclusion and the end of the wash-out period may therefore be explained by the natural fluctuation of symptoms over time in patients with autoimmune pathologies such as pSS. Another limitation of the present study is that the use of a self-assessment questionnaire concerning dry mouth may not be correlated with the global score, which limits the interpretation of the results; an oral quality-of-life questionnaire, such as the OHIP 14 (Slade, 1997) or a questionnaire specific to dry mouth such as the xerostomia inventory (Thomson et al., 1999) would facilitate a better interpretation of the changes.

The comparison of our results concerning the effect of prebiotics on the symptoms of dry mouth is currently not feasible owing to the lack of comparative studies in the scientific literature.

Since the first description of SS by Henrik Sjögren, the most frequent oral complaint of has been reported to be xerostomia, which is associated with significant morbidity and affects the quality of life of patients (Fox et al., 2008; Napeñas and Rouleau, 2014). Moreover, it has been shown that the subjective complaint of xerostomia does not necessarily correlate with the objective measures of hyposalivation (Ramos-Casals et al., 2012; Joanna and Thomson, 2015).

Billing et al. assessed the usefulness of patient-reported xerostomia in the diagnosis of SS by comparing three groups, patients with SS, patient with dry mouth syndrome without SS and patients with incomplete SS (defined by the presence of a focus score > ¼ mm2 or anti-SSA or anti-SSB autoantibodies but not meeting AECG criteria for SS classification). The results of this study indicate that patient-reported xerostomia is highly prevalent in patients with SS and is associated with several clinical phenotypes of this complex syndrome, making it an important indicator of SS. Evidence also suggests that xerostomia is not limited to low salivary flow, but may reflect changes in saliva composition (Billings et al., 2016).

Pijpe et al. performed a longitudinal study, with a mean follow-up period of 3.6 ± 2.3 years, to investigate the loss of salivary gland function in patients with pSS or secondary SS (sSS) in relation to the duration of the disease and use of immunomodulatory drugs as well as the development of subjective complaints over time. They found non-significant decreases in the VAS scores for dry mouth during the day, dry mouth during the night, and difficulty swallowing food without additional liquid in both groups during follow-up. They also reported that patients with a disease duration of less than 1 year had significantly less problems swallowing dry food without liquid than patients with late SS at the start of the study (*p* < 0.05) and that this difference disappeared during follow-up. Moreover, they reported a significant decrease in SSF rates during follow-up in the two groups (*p* < 0.05). On inclusion, patients with early SS exhibited significantly higher SSF rates than those with late SS (*p* < 0.05). After correction for follow-up duration, patients with early-onset SS showed a decrease in stimulated parotid flow rate of 0.02 ml/min in 6 months, whereas those with established and late pSS showed a decrease of 0.01 ml/min in 6 months. No significant difference was found during follow-up for salivary flow rates between treated and untreated patients (Pijpe et al., 2007).

Haldorsen et al. assessed the natural history of exocrine function in a large cohort on the basis of the American-European consensus criteria for SS. The median time from diagnosis to follow-up examination was 5 years. Median USF rates remained unchanged during follow-up. In contrast, high IgG and IgA concentration scores predicted 30% or more worsening of USF at follow-up (Haldorsen et al., 2008).

Another aspect of the present study was the assessment of the salivary microbiota by molecular sequencing to examine the effect of prebiotic and alginate biofilms on the modification of salivary microbiota. Although the *alpha* and *beta* diversity analyses revealed no significant differences in the microbial composition between the study phases, each individual recruited in this study exhibited distinct microbial community composition. Clustering analysis showed that the microbial composition altered only in certain individuals over time, thus, the effect observed is not sufficient to be considered significant. Although the findings indicate that the treatment effect did not substantially alter the microbial composition, it remains likely that a slight change in the relative frequency of a particular species leads to an effect.

By analyzing the two groups of treatment arms separately, it was possible to unravel the subtle changes occurring in the microbiota after each treatment phase. *Lautropia mirabilis*, a bacterium of the human oral cavity and upper respiratory tract (Gerner-Smidt et al., 1994) showed a higher relative abundance at inclusion prior to prebiotic biofilm (A) administration. *Lautropia mirabilis* has been reported to be associated with healthy individuals in comparison with patients with gingivitis, periodontitis or oral squamous cell carcinoma (Abusleme et al., 2013; Zhao et al., 2017; Tsai et al., 2018; Lenartova et al., 2021). A study that characterized the oral microbiota of patients with pSS and control individuals showed that *L. mirabilis* was more abundant in control individuals and patients with pSS who did not experience dry mouth, than in control individuals and patients with pSS with dry mouth (Alam et al., 2020).

The use of sodium alginate biofilm promoted an increase in the abundance of the *Treponema* genus. When the overall effect of sodium alginate biofilm was examined, the species *T. lecithinolyticum* was identified. *Treponema lecithinolyticum* was first described to be strongly associated with disease sites in patients with periodontitis (Wyss et al., 1999). Furthermore, 16S rRNA analyses demonstrated that the presence of *T. lecithinolyticum* was found to be highly correlated with deep pocket depths in patients with generalized aggressive periodontitis and chronic periodontitis (Riep et al., 2009; Griffen et al., 2012) as well as sites with bleeding on probing (Abusleme et al., 2013).

The use of prebiotic biofilm promoted an increase in the relative abundance of the genera *Veillonella* and *Prevotella*. Both *Veillonella* genus and some species of *Prevotella* have been generally associated with periodontal health (Lenartova et al., 2021), and shown to be important pioneer colonizers even at a young age (Könönen et al., 1994; Sulyanto et al., 2019). However, the aciduric and acidogenic genera *Prevotella* and *Veillonella*, respectively, also promote dental caries and gingivitis during hyperglycemia and at high salivary glucose concentrations, typically in patients with type 2 diabetes (Goodson et al., 2017). Several *Veillonella* species have also been reported to facilitate biofilm formation of several *Streptococcus* species (Mashima and Nakazawa, 2014), which in turn, play an important bridging role within the microbial community for the growth and survival of other periodontopathogenic bacteria such as *F. nucleatum* (Zhou et al., 2017). Nevertheless, prebiotic biofilm appeared to stimulate “milder” genera with regard to periodontal infections. Furthermore, pre-treatment with prebiotic biofilm prevented the emergence of the *Treponem*a genus induced by the subsequent treatment with sodium alginate biofilm, suggesting a potential protective effect.

## Data availability statement

The data presented in the study are deposited in the Sequence Read Archive (SRA) data repository, accession number PRJNA925681 (https://www.ncbi.nlm.nih.gov/sra/PRJNA925681).

## Ethics statement

The studies involving human participants were reviewed and approved by the Ethical Review Boards at Brest Hospital approved the protocol of the study was registered with number NCT04206826 in ClinicalTrials.gov. The patients/participants provided their written informed consent to participate in this study.

## Author contributions

MO, SF, and J-OP wrote the manuscript. SB and J-OP designed the work. MO and SF performed acquisition, analysis, or interpretation of data. J-OP, LP, MB-M, and VM revised the manuscript. All authors contributed to the article and approved the submitted version.

## Funding

This study received a grant from Région Bretagne (AAP2016 “Transfert de technologies—chimie, biotechnologies, santé”)—convention 17001696.

## Conflict of interest

The authors declare that the research was conducted in the absence of any commercial or financial relationships that could be construed as a potential conflict of interest.

## Publisher’s note

All claims expressed in this article are solely those of the authors and do not necessarily represent those of their affiliated organizations, or those of the publisher, the editors and the reviewers. Any product that may be evaluated in this article, or claim that may be made by its manufacturer, is not guaranteed or endorsed by the publisher.

## Footnotes

^1^ http://huttenhower.sph.harvard.edu/galaxy/
