# Supplementary figures and images for: Tolerance to intraoral biofilms and their effectiveness in improving mouth dryness and modifying oral microbiota in patients with primary Sjögren’s syndrome: “Predelfi study”
Source: Front Microbiol. 2023 Feb 8;14:1071683. doi: 10.3389/fmicb.2023.1071683 (PMC10245914; doi:10.3389/fmicb.2023.1071683)

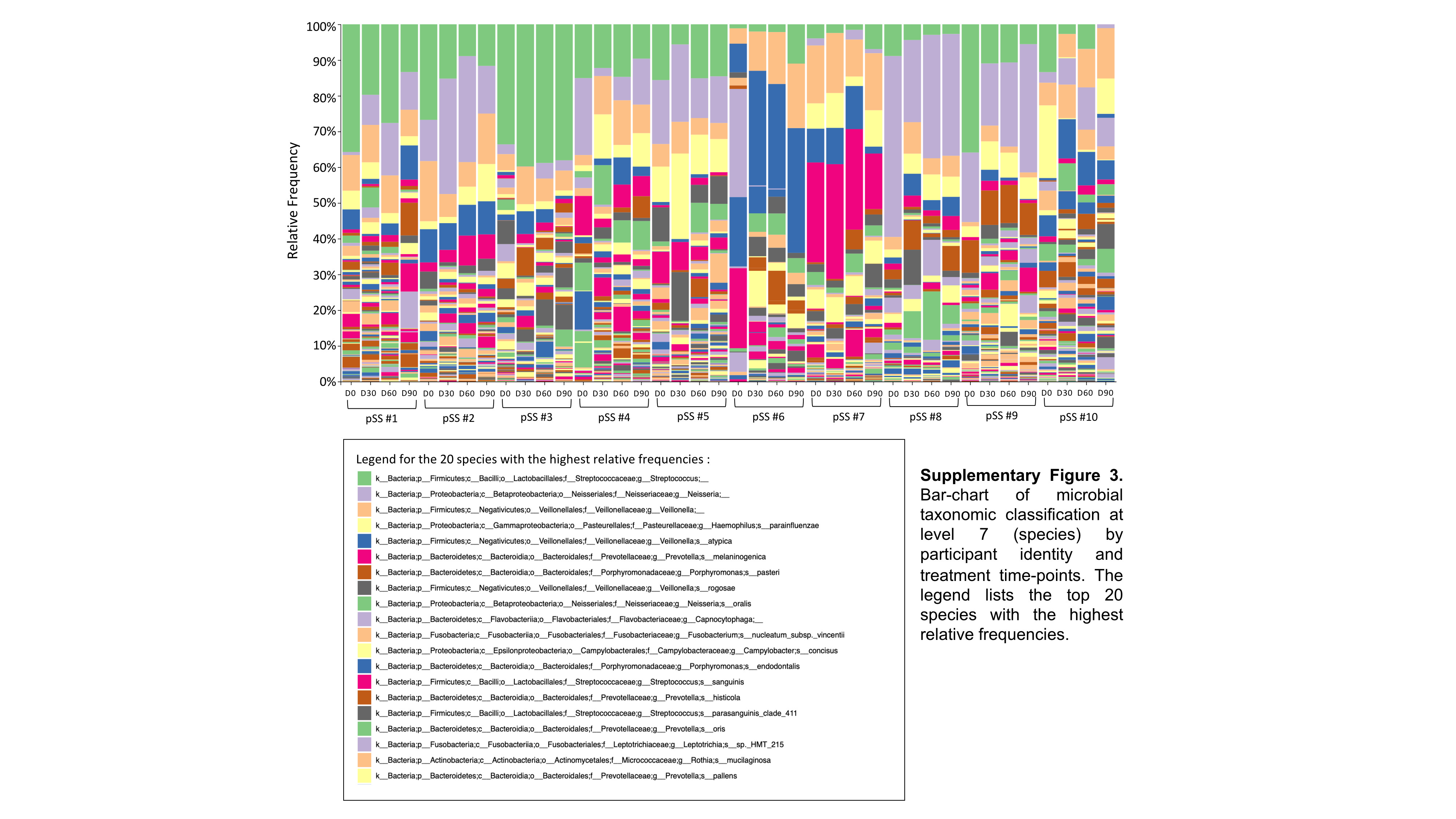

Supplement: Supplementary file 1 [file Image_1.tif]
