# Supplementary material for: Tolerance to intraoral biofilms and their effectiveness in improving mouth dryness and modifying oral microbiota in patients with primary Sjögren’s syndrome: “Predelfi study”
Source: Front Microbiol. 2023 Feb 8;14:1071683. doi: 10.3389/fmicb.2023.1071683 (PMC10245914; doi:10.3389/fmicb.2023.1071683)
Supplement: Supplementary file 4 [file Data_Sheet_1.PDF]

Supplementary Material

1 Supplementary Data

1.1 Supplementary Figures

Supplementary Figure 1 : Flow diagram of the different phases of the Predelfi study.

Supplementary Figure 2: Evolution of the different VAS between the different visits, i.e. mouth dryness, mouth burning sensation, taste alteration, chewing difficulties, swallowing difficulties, and speech difficulty; Patients are separated according each arm: prebiotic biofilm (A) and then sodium alginate biofilm (B) or sodium alginate biofilm (B) and then prebiotic biofilm (A).

Supplementary Figure 3. Bar-chart of microbial taxonomic classification at level 7 (species) by participant identity and treatment time-points. The legend lists the top 20 species with the highest relative frequencies.

1.2 Supplementary Tables

Supplementary Table 1. Self-assessment questionnaire at inclusion

Variable		Population (n=10)
Dry mouth during the day	Never	0
	Sometimes	3 (30%)
	Often	6 (60%)
	Always	1 (10%)
Night awakenings with the obligation to drink to reduce the feeling of dry mouth	Never	4 (40%)
	Sometimes	1 (10%)
	Often	1 (10%)
	Always	4 (40%)
Dental problems (eg difficulty keeping your dental prosthesis in your mouth)	Never	7 (70%)
	Sometimes	3 (30%)
	Often	0
	Always	0
Thick saliva (pasty)	Never	2 (20%)
	Sometimes	6 (60%)
	Often	2 (20%)
	Always	0
Pain in the mouth	Never	5 (50%)
	Sometimes	3 (30%)
	Often	2 (20%)
	Always	0
Spontaneous burning mouth sensation	Never	7 (70%)
	Sometimes	2 (20%)
	Often	0
	Always	1 (10%)

A burning mouth sensation triggered by food	Never	9 (90%)
	Sometimes	0
	Often	1 (10%)
	Always	0
Discomfort when eating acidic foods (eg tomato, cheese, pine nuts, mustard, etc.)	Never	6 (60%)
	Sometimes	4 (40%)
	Often	0
	Always	0
Sore throat when swallowing	Never	5 (50%)
	Sometimes	5 (50%)
	Often	0
	Always	0
The impression that the taste of food changes	Never	6 (60%)
	Sometimes	3 (30%)
	Often	1 (10%)
	Always	0
Halitosis problems	Never	6 (60%)
	Sometimes	3 (30%)
	Often	0
	Always	1 (10%)
Problems chewing and/or swallowing ground or blended foods	Never	8 (80%)
	Sometimes	2 (20%)
	Often	0
	Always	0
Problems chewing and/or swallowing solid foods	Never	5 (50%)
	Sometimes	1 (10%)
	Often	2 (20%)
	Always	2 (20%)
A choking sensation when swallowing	Never	6 (60%)
	Sometimes	2 (20%)
	Often	1 (10%)
	Always	1 (10%)
Need to take sips of liquid to swallow food	Never	3 (30%)
	Sometimes	4 (40%)
	Often	3 (30%)
	Always	0
Difficulty enjoying meals	Never	6 (60%)
	Sometimes	2 (20%)
	Often	2 (20%)
	Always	0
Difficulty carrying on a conversation without stopping to drink	Never	4 (40%)
	Sometimes	4 (40%)
	Often	2 (20%)
	Always	0
Difficulty having social contact in public	Never	8 (80%)

	Sometimes	1 (10%)
	Often	0
	Always	1 (10%)
